# A new mutation with a polycystin phenotypic spectrum in *Caenorhabditis elegans*

**DOI:** 10.17912/W2N37P

**Published:** 2017-11-09

**Authors:** Allyson Whittaker, Gary Schindelman, Shahla Gharib, Paul W. Sternberg

**Affiliations:** 1 Division of Biology and Biological Engineering, Caltech, Pasadena CA 91125

## Description

*lov-1* and *pkd-2*, which encode the *C. elegans* orthologs of human polycystin-1 and -2, are necessary for three particular aspects of male mating behavior. In a screen for male mating defective mutants with similar spectrum of mating defects, we identified a mutation that apparently defines a new locus, *lov-3*.

We isolated the *sy682* mutation in an ethyl-methane sulphonate (EMS)-screen of *Caenorhabditis elegans* strain PS1395 [genotype: *plg-1**(**e2001**d);* *him-5**(**e1490**)]* for mutant males that do not mate efficiently and hence do not form plugs on hermaphrodites (Liu et al, 2017). *sy682* is defective in the males’ response to contact with hermaphrodite and in vulval location (Table 1). The vulval location defect is failing to stop at the vulva. These two phenotypes are associated with *lov-1* (Barr, 1999) and *pkd-2* loss-of-function mutations (Barr et al. 2001; Whittaker et al., 2017). *sy682* maps to the X chromosome and thus is distinct from *lov-1* and *pkd-2*, so it defines a likely new locus, *lov-3*.

**Table:** 

**Genotype**	**Phenotype**	
	**response**	**vulval location**
+/+	47/59	57/57
sy682/sy682	12/61	25/44

**Table 1. Phenotypic analysis of sy682 mutation.** Response, response to contact with hermaphrodite; proportion of males that responded to initial contact with a hermaphrodite. Vulval location, the proportion of males that located the vulva on the first attempt. See Barr & Sternberg (1999) for details

## Reagents

**Strains:PS4770:** *plg-1**(**e2001**d));* *him-5**(**e1490**); sy682***PS6219:** *plg-1**(**e2001**);* *him-5**(**e1490**); sy682*. *sy682* backcrossed 1x**PS1395:** *plg-1**(**e2001**d);* *him-5**(**e1490**)*
